# Molecular targeted and immune checkpoint therapy for advanced hepatocellular carcinoma

**DOI:** 10.1186/s13046-019-1412-8

**Published:** 2019-11-04

**Authors:** Ziyu Liu, Yan Lin, Jinyan Zhang, Yumei Zhang, Yongqiang Li, Zhihui Liu, Qian Li, Ming Luo, Rong Liang, Jiazhou Ye

**Affiliations:** 1 School of Oncology, Guangxi Medical University, Nanning, Guangxi 530021 People’s Republic of China; 2 Department of Medical Oncology, Guangxi Medical University Cancer Hospital, Nanning, Guangxi 530021 People’s Republic of China; 3 Department of Hepatobiliary Surgery, Guangxi Medical University Cancer Hospital, Nanning, Guangxi 530021 People’s Republic of China

**Keywords:** Hepatocellular carcinoma, Molecular targeted therapy, Immunotherapy

## Abstract

Molecular targeted therapy for advanced hepatocellular carcinoma (HCC) has changed markedly. Although sorafenib was used in clinical practice as the first molecular targeted agent in 2007, the SHARPE and Asian-Pacific trials demonstrated that sorafenib only improved overall survival (OS) by approximately 3 months in patients with advanced HCC compared with placebo. Molecular targeted agents were developed during the 10-year period from 2007 to 2016, but every test of these agents from phase II or phase III clinical trial failed due to a low response rate and high toxicity. In the 2 years after, 2017 through 2018, four successful novel drugs emerged from clinical trials for clinical use. As recommended by updated Barcelona Clinical Liver cancer (BCLC) treatment algorithms, lenvatinib is now feasible as an alternative to sorafenib as a first-line treatment for advanced HCC. Regorafenib, cabozantinib, and ramucirumab are appropriate supplements for sorafenib as second-line treatment for patients with advanced HCC who are resistant, show progression or do not tolerate sorafenib. In addition, with promising outcomes in phase II trials, immune PD-1/PD-L1 checkpoint inhibitors nivolumab and pembrolizumab have been applied for HCC treatment. Despite phase III trials for nivolumab and pembrolizumab, the primary endpoints of improved OS were not statistically significant, immune PD-1/PD-L1 checkpoint therapy remains to be further investigated. This review summarizes the development and progression of molecular targeted and immune-based checkpoint therapies in HCC.

## Introduction

**Fig. 1:**
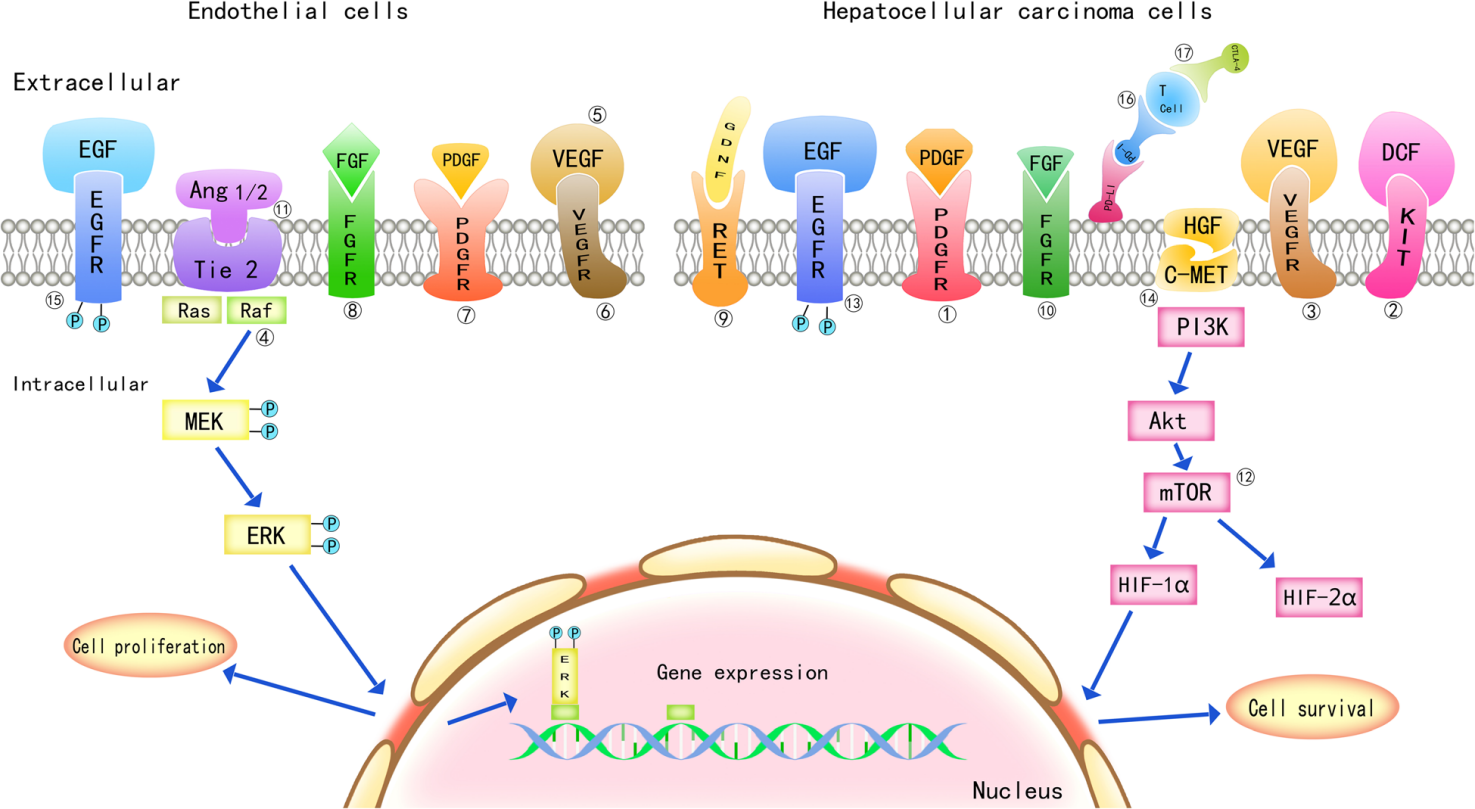
Important target molecules and signal transduction pathways in hepatocarcinogenesis and progression. Drug-targeting receptors are present on the cell membrane of hepatoma cells and endothelial cells. EGFR: epidermal growth factor receptor; Tie2: angiopoietin receptor; FGFR: fibroblast growth factor receptor; PDGFR: platelet-derived growth factor receptor; VEGFR: vascular endothelial growth factor receptor; RET: glial cell-derived neurotrophic factor receptor; C-MET: hepatocyte growth factor receptor; KIT: stem cell factor receptor. Two signal transduction pathways, Ras/Raf/MEK/ERK and PI3K/Akt/mTOR/HIF, affect the proliferation and survival of HCC cells by regulating gene expression

Hepatocellular carcinoma (HCC) is the sixth most common malignancy and the fourth leading cause of cancer-related death worldwide [1]. Because the symptoms of early HCC are often inconspicuous, most patients are diagnosed at an advanced stage, eliminating the possibility of local treatment, such as curative hepatic resection, tumor ablation or transtarterial therapy. Therefore, the systematic treatment of advanced HCC is of great concern. Since sorafenib was approved as the first small oral molecular targeted medicine for patients with advanced-stage HCC in 2007, molecular targeted therapy for advanced HCC has changed markedly. However, although the SHARPE trial (in Europe and USA) [2] and the Asian-Pacific study (in Asia-Pacific regions) [3] demonstrated that sorafenib significantly improved the survival benefit for patients with advanced HCC, anticancer efficacy remains unsatisfactory because sorafenib only prolongs the overall survival (OS) period by approximately 3 months compared with placebo. From 2007 to 2016, various molecular targeted drugs for advanced HCC were developed (Fig. 1, Table 1). However, most of the phase II or III clinical trials for these medicines failed, as the results did not show that these drugs achieved a better survival benefit for advanced HCC patients compared with sorafenib or they were not well tolerated with severe adverse events. Fortunately, there has been substantial progress in testing new and efficacious systemic therapies for patients with an advanced-stage HCC, with six new agents exhibiting clinical efficacy in phase 3 trials in the past 2 years. Lenvatinib has successfully become the first-line treatment in clinical practice, and regorafenib, cabozantinib, and ramucirumab have been recommended as second-line treatment options. In addition, the clinical benefits of immune-based therapies for HCC have been emerging. In a single-group phase 1/2 trial [4], the novel programmed cell death 1 (PD-1) checkpoint inhibitor nivolumab resulted in promising survival in patients who had disease progression or unacceptable adverse effects with sorafenib, which prompted FDA approval under the accelerated program. In contrast, another PD-1 inhibitor, pembrolizumab, for second-line treatment did not confer longer OS or progression-free survival (PFS) compared to placebo in a recently reported phase III trial [5]. This review summarizes the development and progression of molecular targeted and immune-based checkpoint therapies in HCC.

**Table 1:** Clinical research on molecular targeted drugs for hepatocellular carcinoma

Drug	Study phase	Trial name	Publication time	Combination	Versus	Fist author	Result	Primary end point	Targets/Pathway
First-line systemic therapy									
Sorafenib	III	SHARP	2008	/	placebo	M. Llovet	Positive	OS: 10.7 months	VEGFR1/2/3; PDGFR;Ras/Raf/Mek/Erk
	III	ORIENTAL	2009	/	placebo	Ann-Lii Cheng	Positive	OS: 6.5 months	
Lenvatinib	II	/	2017	/	single-arm	Kenji Ikeda	Positive	OS: 18.7 months	VEGFR1/2/3; FGFR1/2/3/4; FGF; PDGFR2; RET
	III	REFLECT	2018	/	sorafenib	Masatoshi	Positive	OS: 13.6 months PFS: 8.9 months	
Second-line systemic therapy									
Multitarget tyrosine inhibitor									
Regorafenib	II	/	2013	/	single-arm	Jordi Bruix	Positive	OS: 13.8 months	VEGFR1; RETRAF1; TIE-2; BRAF; PDGFR; FGFR;
	III	RESORCE	2017	/	placebo	Jordi Bruix	Positive	OS: 10.6 months	
Cabozantinib	II	/	2017	/	placebo	R.K. Kelley	Positive	OS: 11.5 months	c-Met; VEGFR1/2/3
	III	CELESTIAL	2018	/	placebo	Abou-Alfa	Positive	OS: 10.2 months PFS: 5.2 months	
VEGF receptor inhibitor									
Ramucirumab	III	REACH	2015	/	placebo	Andrew X Zhu	Negative	OS: 9.2 months	VEGFR2
	III	REACH-2	2019	/	placebo	Andrew X Zhu	Positive	OS: 8.5 months	
Anti-PD-1 antibody									
Nivolumab	I/II	CheckMate −040	2017	/	single-arm	El-Khoueiry	Positive	OS: 15 months	PD-1
Pembrolizumab	II	KEYNOTE − 242	2018	/	single-arm	Andrew X Zhu	Positive	OS: 12.9 months	PD-1
Other targeted therapies									
Antiangiogenic drugs									
Bevacizumab	II	/	2006	GEMOX	single-arm	Andrew X Zhu	Positive	OS: 9.6 months	VEGF
Brivanib	II	/	2011	/	single-arm	JW Park	Positive	OS: 10 months PFS: 2.7 months	VEGF; FGFR
	III	BRISK-PS	2013	/	placebo	M. Llovet	Negative	OS: 9.4 months	
	III	BRISK-FL	2013	/	sorafenib	PJ. Johnson	Negative	OS: 9.5 months	
Linifanib	II	/	2013	/	single-arm	Han Chong	Positive	OS: 9.7 months PFS: 3.7 months	VEGF; PDGFR
	III	LiGHT	2015	/	sorafenib	Calin Cainap	Negative	OS: 9.1 months TTP: 5.4 months	
Sunitinib	II	/	2009	/	single-arm	Andrew X Zhu	Positive	OS: 9.8 months PFS: 3.9 months	VEGFR; PDGFRa/b; c-Kit;
	III	SUN1170	2013	/	sorafenib	Ann-Lii Cheng	Negative	OS: 7.9 months PFS: 3.6 months	
Immunoreactive drugs									
Tremelimumab	I	/	2017	ablation	single-arm	Duffy AG	Positive	OS: 12.3 months TTP: 7.4 months	CTLA-4
	II	/	2013	/	single-arm	Sangro B	Positive	OS: 8.2 months TTP: 6.5 months	
Drugs targeting EGFR									
Erlotinib	II	/	2018	bevacizumab	sorafenib	Thomas	Negative	OS: 8.55 months PFS: 4.37 months	EGFR
	III	SEARCH	2015	sorafenib	placebo	Andrew X Zhu	Negative	OS: 9.5 months	
Cetuximab	II	/	2008	GEMOX	single-arm	Asnacios	Positive	OS: 9.5 months PFS: 4.7 months	EGFR
	II	/	2010	/	single-arm	Andrew X Zhu	Negative	OS: 9.6 months PFS: 1.4 months	
	II	/	2011	XELOX	single-arm	Sanoff	Negative	TTP: 4.5 months DCR: 83%	
Lapatinib	II	/	2009	/	single-arm	TB-Saab	Negative	OS: 12.6 months PFS: 1.9 months	EGFR
Drugs targeting PI3K/Akt/mTOR signal pathway									
Sirolimus	I	/	2008	/	single-arm	Magnus Rizell	Positive	OS: 6.5 months	PI3K/Akt/mTOR
	II	/	2012	/	single-arm	Thomas	Positive	OS: 6.6 months PFS: 3.8 months	
Everolimus	I/II	/	2011	/	single-arm	Andrew X Zhu	Negative	OS: 8.4 months	PI3K/Akt/mTOR
	III	EVOLVE-1	2014	/	placebo	Andrew X Zhu	Negative	OS: 7.6 months PFS: 3.0 months	
C-Met inhibitor									
Tivantinib	II	/	2013	/	placebo	Santoro	Positive	PFS: 2.7 months	C-Met
	III	METIV-HCC	2018	/	placebo	Rimassa	Negative	OS: 8.4 months	

*PFS* Progression-free survival, *OS* Overall survival, *DCR* Disease control rate. *EGFR* Epidermal growth factor receptor, *Tie2* Angiopoietin receptor, *FGFR* Fibroblast growth factor receptor, *PDGFR* Platelet-derived growth factor receptor, *VEGFR* Vascular endothelial growth factor receptor, *RET* Glial cell-derived neurotrophic factor receptor, *C-MET* Hepatocyte growth factor receptor, *KIT* Stem cell factor receptor

## First-line systemic therapy

### Sorafenib

Sorafenib is an oral small molecule multikinase inhibitor that exerts an anticancer effect by simultaneously suppressing angiogenesis via inhibition of vascular endothelial growth factor receptor (VEGFR-1,2,3) and platelet-derived growth factor receptor (PDGFR) and the growth of tumor cells directly through downregulation of the Ras/Raf/Mek/Erk signaling pathway [6, 7].

In 2007, two phase III randomized, multicenter, double-blind, placebo-controlled trials, the SHARP trial (in Europe and the USA) [2] and ORIENTAL trial (in Asia-Pacific regions) [3], reported promising results that sorafenib significantly increased survival for advanced HCC patients with different territories when compared with placebo. The SHARP trial enrolled 602 advanced HCC patients in northern America and western Europe, and the results demonstrated that the survival benefits from sorafenib were superior to placebo. The median OS was 10.7 months in the sorafenib group (a dose of 400 mg twice daily) and 7.9 months in the placebo group. The ORIENTAL trial enrolled advanced HCC 271 patients from the Asia-Pacific region and reported a magnitude of survival benefit similar to that of the SHARP trial. The median OS was 6.5 months in patients treated with sorafenib (a dose of 400 mg twice daily) compared with 4.2 months in those who received placebo. Based on the results from the SHARP and ORIENTAL trials, sorafenib was approved by the US FDA and EMEA for advanced HCC systematic treatment. Furthermore, in 2010, sorafenib was recommended by Barcelona Clinical Liver Cancer (BCLC) treatment algorithms [8] and version 1.2008 NCCN guidelines [9] as a first-line targeted molecular therapy for advanced HCC globally. Nonetheless, the SHARP and ORIENTAL trials reported outcomes that sorafenib only prolongs the OS period by approximately 3 months in patients with advanced HCC. Systemic therapy for advanced HCC has developed markedly since sorafenib was applied to the treatment for advanced HCC in 2007. Although many agents were developed between 2007 and 2016, most of them failed in clinical trials, and rare molecular drugs have become the 1st line and 2nd line systemic treatments for advanced HCC in clinical practice.

### Lenvatinib

Lenvatinib is another oral small molecule multikinase inhibitor that selectively inhibits tyrosine kinases (e.g., VEGFR1, VEGFR2, VEGFR3), fibroblast growth factor receptor (FGFR1, FGFR2, FGFR3, FGFR4), PDGFR2, FGF and RET to suppress tumor angiogenesis and growth [10]. Lenvatinib has been certified to invoke strong antiangiogenic and anticancer effects and has been approved for the treatment of differentiated thyroid carcinoma [11]. The phase II trial [12] of lenvatinib for the treatment of patients with advanced HCC demonstrated that 12-mg QD of the agent had significant survival benefits, with a disease control rate (DCR) of 78% and a median OS of 18.7 months, as well as acceptable toxicity profiles without severe adverse events. A phase III randomized, multicenter, open-label, non-inferiority trial, the REFLECT trial [13] enrolled 954 patients and compared the efficacy of lenvatinib versus sorafenib for first-line treatment of patients with unresectable HCC. The results presented a positive outcome, whereby lenvatinib achieved a better OS benefit than did sorafenib. The median OS duration was 13.6 months for 478 patients in the lenvatinib group (12 mg/day for bodyweight ≥60 kg or 8 mg/day for bodyweight < 60 kg) compared with 12.3 months for 476 patients in the sorafenib group (400 mg twice-daily). In OS subanalysis, patients were stratified by race (Asian or non-Asian), vascular invasion and/or EHS (yes or no), Eastern Cooperative Oncology Group performance status (PS) (0 or 1), and body weight (< 60 kg or ≥ 60 kg), and lenvatinib also resulted in longer OS than sorafenib in almost all subgroups. In particular, patients in the serum AFP level > 200 ng/mL group treated with lenvatinib had significantly longer OS than did those treated with sorafenib (10.4 months vs 8.2 months). In addition, the REFLECT trial demonstrated a statistically significant improvement compared with sorafenib with respect to all secondary efficacy endpoints, such as PFS, TTP, and ORR. The median PFS for lenvatinib was longer than that for sorafenib, and the median time to progression was 8.9 months (95% CI 7.4–9.2) for patients in the lenvatinib group compared to 3.7 months (3.6–5.4) for patients in the sorafenib group. On August 16, 2018, the US FDA officially approved lenvatinib for advanced HCC systematic treatment. Moreover, lenvatinib is recommended by the version 2.2019 NCCN guidelines [14] as the second first-line targeted molecular treatment for advanced HCC.

## Second-line systemic therapy

### Multitarget tyrosine inhibitors

#### Regorafenib

Regorafenib is a small molecule multitarget inhibitor of VEGFR1, TIE-2, RETRAF-1, BRAF, PDGFR, FGFR, and CSF1R. In 2013, a multicenter, open-label and phase II clinical trial [15] revealed that the use of regorafenib as a second-line therapy during the progression of intermediate and advanced HCC after sorafenib treatment failure achieved promising therapeutic outcomes, with a DCR of 72% and an OS of 13.8 months, providing evidence of antitumor activity in patients with intermediate or advanced HCC that progressed following first-line sorafenib treatment. The RESORCE study [16] led by Bruix J, a randomized, double-blind, placebo-controlled, phase 3 trial, enrolled 573 patients with HCC who tolerated and progressed on sorafenib (≥400 mg/day for ≥20 of last 28 days of treatment) from 152 medical institutions in 21 countries. The participants were randomly assigned by 2:1 to a regorafenib (oral dose 160 mg daily during weeks 1–3 of each 4-week cycle) or placebo-control (once daily during weeks 1–3 of each 4-week cycle) group. The results showed that the median OS of the regorafenib group was 10.6 months, which was significantly superior to the 7.8 months observed in the placebo-control group. Considering the promising evidence from the RESORCE study, on December 22, 2017, US FDA officially approved regorafenib for use in patients with HCC. Furthermore, the version 1.2017 NCCN guidelines [17] recommend regorafenib as a second-line agent for HCC who progress on sorafenib treatment.

#### Cabozantinib

Cabozantinib is a tyrosine kinase inhibitor of c-Met, AXL, VEGFR1, − 2, and − 3. A phase II trial [18] placebo-controlled randomized discontinuation study of cabozantinib in HCC patients led by R. K. Kelley randomized 12 patients to placebo and 10 to cabozantinib. The results revealed a median week 12 PFS of 5.2 months and a median week 12 OS of 11.5 months for cabozantinib, indicating good antitumor activity in HCC. Abou-Alfa et al. then carried out a randomized, double-blind, phase III clinical study [19] to evaluate cabozantinib as a second-line therapy for advanced HCC and the development of resistance to sorafenib, in which 707 patients were randomly assigned in a 2:1 ratio to receive cabozantinib (60 mg once daily) or matching placebo. The results showed significantly longer OS and PFS with cabozantinib than with placebo. The median OS and PFS were 10.2 months and 5.2 months in the cabozantinib group compared with 8.0 months and 1.9 months in the placebo group. Cabozantinib has been approved by the US FDA for the treatment of HCC. Furthermore, the NCCN guidelines [14] (version 2.2019) recommend cabozantinib as a second-line agent for HCC patients who progress on sorafenib, offering an alternative option for second-line treatment of HCC.

### VEGF receptor inhibitors

#### Ramucirumab

Ramucirumab is a recombinant IgG1 monoclonal antibody and VEGF receptor-2 antagonist that has been approved by the US FDA for the treatment of gastric cancer [20] (on April 21, 2014), non-small cell lung cancer (on December 12, 2014) and colorectal cancer [21] (on April 29, 2015). The REACH trial was a randomized, double-blind, multicenter (154 centers in 27 countries), phase 3 trial [22] led by Andrew X. Zhu in 2010 to investigate ramucirumab versus placebo as a second-line treatment in 565 patients with advanced progressing HCC following first-line therapy with sorafenib. The results showed an OS of 9.2 months in the ramucirumab group (8 mg/kg every 2 weeks) compared with 7.6 months in the placebo group. Although the OS periods between the two groups were not statistically significant, in subgroup analysis, patients with elevated baseline serum AFP concentrations of 400 ng/mL or greater achieved a better OS benefit from ramucirumab compared with placebo. The median OS in the ramucirumab group was 7.8 months, which was significantly greater than the 4.2 months in the placebo-control group. Based on this finding, the REACH-2 study [23], a randomized, double-blind, placebo-controlled, phase 3 trial also led by Andrew X. Zhu, was consequently conducted in 2015 with 292 patients with advanced HCC and α-fetoprotein concentrations of 400 ng/mL or higher from 92 hospitals, clinics, and medical centers in 20 countries. Among the patients, 197 were randomly assigned to the ramucirumab group and 95 to the placebo group. According to the results, the OS period was 8.5 months in the ramucirumab group (8 mg/kg every 2 weeks) compared with 7.3 months in the placebo group, and the median PFS was significantly increased in the ramucirumab group (2.8 months) compared with the placebo group (1.6 months), though the proportion of patients’ ORR was not significantly different between the groups. In addition, ramucirumab was well tolerated with a low incidence of adverse events and a manageable safety profile. Considering that the REACH-2 study confirmed the result of subgroup analysis in the REACH trial, which is the first positive phase III trial performed in a biomarker-selected patient population with advanced HCC, on May 10, 2019, the FDA approved ramucirumab as a single agent for HCC in patients who have an AFP ≥400 ng/mL and have been previously treated with sorafenib. Ramucirumab is also recommended by the NCCN guideline [14] (version 2.2019) as a potential well-tolerated second-line treatment for patients with advanced HCC and elevated AFP levels.

### Anti-PD-1 antibody

#### Nivolumab

Nivolumab, a PD-1 inhibitor, was approved by the FDA in 2017 as a second-line treatment for advanced HCC in the presence of sorafenib resistance. A phase 1/2, open-label, non-comparative, dose escalation and expansion trial [4] (CheckMate 040) led by El-Khoueiry AB was conducted to assess the safety and efficacy of nivolumab as a first-line therapy in patients with advanced HCC. The results revealed that in the dose-escalation phase, the overall objective response rate was 15%, with a DCR of 58% and an OS of 15 months. In the dose-expansion phase, more than 200 patients who were treated with nivolumab had a six-month survival rate of 83% and a nine-month survival rate of 74%. The study also shows that nivolumb has a manageable safety profile. On June 24, 2019, the Bristol-Myers Squibb Company published a phase 3 randomized, multicenter study evaluating opdivo (nivolumab) versus sorafenib as a first-line treatment in patients with unresectable HCC. However, per the pre-specified analysis, statistical significance for its primary endpoint of OS was not achieved, and the specific data have not been published. Regardless, the trial CheckMate-459 revealed a clear trend of improvement in OS for patients treated with opdivo compared to sorafenib, and exploration of opdivo in HCC will continue.

#### Pembrolizumab

Pembrolizumab is a recombinant monoclonal human immunoglobulin IgG4 antibody specific for the human PD-1 checkpoint. The FDA approved pembrolizumab for the treatment of patients with unresectable or metastatic melanoma in 2019. A non-randomized, multicenter, open-label phase II study [24] (KEYNOTE-224) led by Andrew X Zhu was performed to assess the efficacy and safety of pembrolizumab in patients with advanced HCC. In this study, participants received 200 mg pembrolizumab intravenously every 3 weeks for approximately 2 years or until disease progression or unacceptable toxicity. The results showed a complete response rate of 1%, partial response rate of 16%, and stable rate of 44%. Tumor remission rates of 17% and DCRs of more than 60% were achieved with pembrolizumab in patients with advanced HCC and were maintained for a long period of time, with a median OS of 12.9 months. In addition, pembrolizumab was well tolerated with few adverse reactions. Considering that pembrolizumab is effective and tolerable in patients with advanced HCC who had previously been treated with sorafenib, the US FDA has approved the priority review application for pembrolizumab for the indication of a second-line treatment for HCC in 2019 and progressed the KEYNOTE-240 trial [5] (a randomized, placebo-controlled phase III study of pembrolizumab vs best support care in patients with previously treated advanced HCC). Unfortunately, failure was declared for the KEYNOTE-240 trial 3 months later. Although the significance of the trial did not reach the prespecified statistical criteria, pembrolizumab reduced the risk of death by 22% and improved PFS compared with placebo. Additionally, the ORR in the pembrolizumab arm was consistent with that of KEYNOTE-224, and the safety profile was comparable to that established for pembrolizumab monotherapy. These results are consistent with KEYNOTE-224, further supporting pembrolizumab as second-line treatment for HCC patients.

## Other targeted therapies

### Antiangiogenic drugs

Because angiogenesis has been demonstrated to be a major mechanism contributing to malignant tumor growth and metastasis, antiangiogenic drugs have become an important strategy for the systematic treatment of cancers, particularly for HCC, which is a typical blood-rich tumor overexpressing various angiogenic factors.

#### Bevacizumab

Bevacizumab is a monoclonal antibody against VEGF. When specifically binding to VEGF, it prevents VEGF from interacting with VEGF receptors on the surface of endothelial cells (Flt-1 and KDR) and blocks the VEGF-mediated pathway, which leads to suppression of vascular endothelial cell proliferation and tumor angiogenesis. The combination of bevacizumab and atezolizumab has been approved for the treatment of renal clear cell carcinoma and the combination of bevacizumab and carboplatin for non-small cell lung cancer by the US FDA [25]. However, bevacizumab has not been approved for use in the treatment of HCC. In its phase II trial [26], bevacizumab exhibited significant clinical and biologic activity in nonmetastatic HCC, with an objective response rate of 13%, a 6-month PFS of 65%, a median PFS of 6.9 months, and an OS of 53% at 1 year, 28% at 2 years, and 23% at 3 years; however, severe adverse events such as bleeding, leukopenia/neutropenia, transient elevation of aminotransferases and hypertension occurred. No phase III trial of bevacizumab for HCC has been conducted to date. Another phase II study [27] led by Andrew X. Zhu showed that a combination of gemcitabine, oxaliplatin and bevacizumab (GEMOX-B regime, in which for cycle 1 (14 days), 10 mg/kg bevacizumab was administered alone intravenously on day 1. For cycle 2 and thereafter (28 days/cycle), bevacizumab 10 mg/kg was administered on days 1 and 15; gemcitabine 1000 mg/m2 was administered as a dose rate infusion of 10 mg/m2/min followed by oxaliplatin at 85 mg/m2 on days 2 and 16) achieved a certain effect for advanced HCC patients, with a median OS and PFS of 9.6 and 5.3 months, respectively. The GEMOX-B regime was safely administered with close monitoring and demonstrated moderate antitumor activity for patients with advanced HCC. A phase II trial of bevacizumab + erlotinib vs. sorafenib (*clinicaltial.gov**,* No. NCT00881751) for the treatment of unresectable HCC is currently underway.

#### Brivanib

Brivanib is a selective dual inhibitor of VEGF and FGFR, suppressing angiogenesis and tumor cell growth. Encouraging antitumor activity has been shown in preclinical and phase I trials [28]. In a phase II, an open-label study [29] of brivanib as a first-line therapy in patients with advanced HCC, oral administration at a dose of 800 mg once daily showed good antitumor activity, with a six-month DFS rate of 18.2%, a median PFS of 2.7 months and a median OS of 10 months. Moreover, brivanib was generally well tolerated. However, the results of the subsequent randomized phase III BRISK-FL study [30] were not satisfactory, with a primary end point of OS in the brivanib-treating group that was not superior to that in the sorafenib-treated group. The median OS was 9.9 months for sorafenib and 9.5 months for brivanib. Second end point data of TTP, ORR and DCR were similar to those of sorafenib. Another multicenter, double-blind, randomized, placebo-controlled BRISK-PS study [31] showed that brivanib did not significantly improve OS compared with placebo in patients with advanced HCC and who were treated with and intolerant to sorafenib. The median OS was 9.4 months for brivanib treatment and 8.4 months for placebo treatment. Both the BRISK-FL and BRISK-PS studies failed, suggesting that brivanib does not present promising antitumor activity in advanced HCC.

#### Linifanib

Linifanib is a tyrosine kinase inhibitor of VEGF and PDGFR. In the phase II trial [32] led by Han Chong Toh, administration of single-agent linifanib orally at a fasting dose of 0.25 mg/kg daily to patients with Child-Pugh class A hepatic function and every other day to patients with Child-Pugh class B hepatic function showed promising clinical activity in patients with advanced HCC, with a median PFS of 3.7 months and a median OS of 9.7 months. As acceptable safety profile was also reported. In an open-label randomized phase III trial [33] conducted by Calin Cainap to evaluate the efficacy and tolerability of linifanib versus sorafenib in patients with advanced HCC, 1035 patients were randomly assigned in a 1:1 ratio to linifanib 17.5 mg once daily or sorafenib 400 mg twice daily. The median OS was 9.1 months in the linifanib group and 9.8 months in the sorafenib group, suggesting that linifanib and sorafenib have similar OS in advanced HCC. Indeed, the redefined superiority and noninferiority OS boundaries were not met for linifanib, and the primary end point was not reached. In addition, safety results favored sorafenib.

#### Sunitinib

Sunitinib is a small molecule tyrosine kinase inhibitor of VEGFR, PDGFR-a/b, c-Kit, FLT3 and RETS. The antitumor activity of sunitinib was observed in a phase II clinical trial [34] led by Andrew X. Zhu. The median OS and PFS were 9.8 months and 3.9 months, respectively, and blood vessel permeability and levels of circulating inflammatory biomarkers were altered after treatment. Sunitinib can rapidly reduce vascular leakage, especially in patients with relatively slow progression. In an open-label, phase III trial [35] evaluating whether sunitinib is superior or equivalent to sorafenib in advanced HCC, 1074 patients were stratified and randomly assigned 1:1 to receive sunitinib 37.5 mg once per day or sorafenib 400 mg twice per day. The results for sunitinib and sorafenib were as follows: median OS of 7.9 versus 10.2 months, median PFS of 3.6 versus 3.0 months, and TTP of 4.1 versus 3.8 months. Sunitinib failed to provide better anticancer activity than sorafenib but was associated with more frequent and severe adverse events (AEs). Therefore, the trial was halted in 2010. Nevertheless, sunitinib is still used in treatment for liver fibrosis due to its antiangiogenic and fibrosis inhibitory properties [36].

### Immunoreactive drugs

Ipilimumab was the first real immunoreactive drug to be used clinically, and immuno-targeted medicines for malignant tumor therapy have since developed rapidly. With the recent success of checkpoint inhibitors in multiple tumors, their role in HCC has also been explored, and benefits of other immunotargeting agents can be expected when the immune checkpoint inhibitors nivolumab and pembrolizumab become available for HCC treatment.

#### Tremelimumab

Tremelimumab is a fully human monoclonal antibody that binds to the cytotoxic T lymphocyte-associated antigen 4 (CTLA-4) on the surface of activated T lymphocytes. A pilot clinical study [37] conducted by Bruon evaluated the antitumor and antiviral effects of tremelimumab in patients with advanced HCC and chronic HCV infection. After oral administration of a dose of 15 mg/kg IV every 90 days, promising anticancer and antiviral effects with a partial response rate of 17.6%, a disease control rate of 76.4%, time to progression of 6.48 months, and a significant decline in viral load, were observed. These findings suggest that tremelimumab immunotherapy is a promising treatment option, in particular for inhibiting the progression of hepatitis C-related advanced HCC. Tremelimumab is safe because treatment is mostly well tolerated in patients, with only a few experience disabling AEs. No patient received systemic steroids, and there were no treatment-related deaths. Another study [38] conducted by Duffy in which patients with advanced HCC were treated with tremelimumab (3.5 or 10 mg/kg IV every 4 weeks for a total of 6 doses) in combination with an ablative procedure performed during week 6. The results show that ablative therapy induced a peripheral immune response, possibly enhancing the effect of tremelimumab in patients with advanced HCC. Six- and 12-month probabilities of tumor PFS for this refractory HCC population were 57.1 and 33.1%, respectively, with a median time to TTP of 7.4 months and a median OS of 12.3 months. In addition, six-week tumor biopsies showed a clear increase in CD8+ T cells only in patients presenting a clinical benefit. These two studies suggested that tremelimumab treatment of patients with advanced HCC is feasible and leads to the accumulation of intratumoral CD8+ T cells and possibly surrogate reductions in HCV viral load.

### Drugs targeting EGFR

It is clear that overexpression of epidermal growth factor receptor (EGFR) on the membrane of HCC cells significantly promotes HCC tumorigenesis and progression. Additionally, upon ligand (EGF and TGF) binding, EGFR activates tyrosine kinases on the cell surface, which leads to the disordered growth of hepatoma cells. As a target for the treatment of HCC, several small molecule EGFR inhibitors have been developed.

#### Erlotinib

Erlotinib is a tyrosine kinase inhibitor that specifically targets EGFR and EGF-1. Several studies have shown that erlotinib has good anticancer activity in non-small cell lung cancer [39] and pancreatic cancer [40]. A phase II trial [41] led by Thomas revealed that single-agent erlotinib (oral dose 150 mg daily for 28-day cycles) is well tolerated with a modest disease-control benefit in HCC, as manifested as moderately prolonged PFS and OS when compared with historical controls. Another phase III, randomized, double-blind, placebo-controlled trial [42] led by Andrew X. Zhu revealed that erlotinib associated with sorafenib did not improve survival when compared with sorafenib plus placebo in patients with advanced HCC. The median OS in patients treated with erlotinib associated with sorafenib was 9.5 months, whereas the median OS of patients treated with sorafenib plus placebo was 8.5 months. Therefore, the efficacy of erlotinib in liver cancer remains to be further studied.

#### Cetuximab

Cetuximab is an IgG1 monoclonal antibody against EGFR that can specifically bind to EGFR on various tumor cells and inhibit the binding of other ligands, thereby suppressing tumor growth and progression. Cetuximab has been approved by the FDA as a first-line treatment for advanced colorectal cancer [43] and advanced head and neck cancer [44], with promising survival benefits. Unfortunately, a phase II trial [45] showed that although cetuximab could be safely administered with tolerable toxicity profiles, it exhibited no antitumor activity in HCC. Another phase II study [46], indicated that the combination of cetuximab (a dose of 400 mg/m2 initially then 250 mg/m2 weekly) and gemcitabine plus oxaliplatin (GEMOX regimen) failed to provide therapeutic effects comparable to that of either single-use cetuximab or GEMOX. Another phase II trial [47] led by Sanoff et al. showed that the combination of cetuximab and capecitabine plus oxaliplatin (capecitabine 850 mg/m2 bid days 1–14, oxaliplatin 130 mg/m2 day 1, and cetuximab 400 mg/m2 day 1 then 250 mg/m2 weekly for each 21 day cycle) in advanced HCC resulted in a DCR of 83%, median TTP of 4.5 months and OS of 4.4 months. This result suggested that the time to progression and OS were shorter than would be expected for treatment with sorafenib.

#### Lapatinib

Lapatinib is another small molecule tyrosine kinase inhibitor of EGF that can effectively inhibit the ATP checkpoint and prevent homogenization and heterodimerization between EGFR and HER2, which can inhibit tumor cell growth. A multi-institutional phase II trial [48] conducted by Bekaii determined the safety and efficacy of lapatinib in advanced HCC. Interestingly, tumor and blood specimens were analyzed for expression of HER2/NEU/CEP17 and downstream signaling pathway protein status. After an oral dose of lapatinib of 1500 mg/day in 28-day cycles, the median PFS was 1.9 months, and the median OS was 12.6 months. However, somatic mutations in EGFR (exons 18–21) and HER2/NEU were not found. In addition, PTEN, P-AKT, andP70S6 K expression did not correlate with survival. Overall, the results suggest that lapatinib is well tolerated and that only a subgroup of patients obtain a benefit, among whom the predictive molecular and clinical characteristics have not yet fully been defined.

### Drugs targeting the PI3K/Akt/mTOR signaling pathway

The PI3K/Akt/mTOR signaling pathway plays an important role in HCC tumorigenesis and progression. PI3K regulates the proliferation, growth, survival and angiogenesis of tumor cells. Activated PI3K phosphorylates and activates AKT, which is localized in the plasma membrane. AKT transmits the signal to downstream targets and then activates mTOR. Blocking this signaling pathway, particularly inhibition of mTOR activation, may specifically suppress tumor cell growth.

#### Sirolimus

Sirolimus is an inhibitor of mTOR [49]. By inhibiting expression of hypoxia inducible factor-1 α and decreasing the synthesis and secretion of VEGF, then effectively inhibits angiogenesis and HCC proliferation. The PFS and OS of patients after treatment with rapamycin analogs (sirolimus) was 15.3 weeks and 26.4 weeks in a phase II trial [50] led by Decaens. One patient achieved a complete response (CR), with 8 having stable disease (SD); a median OS of 6.5 months was reported in another trial led by Rizell [51]. Both studies suggest that first-line sirolimus has antitumoural efficacy in advanced HCC. However, there are no reports of phase III trials of sirolimus, and larger trials with Child-Pugh A patients are needed.

#### Everolimus

Everolimus is a rapalog and inhibitor of mTOR that has been approved for the treatment of renal and breast cancer. The results from a phase I/II clinical trial [52] preliminarily revealed the curative effect of everolimus for HCC, with a median OS and PFS of 8.4 and 3.8 months, respectively. Considering the different targets of everolimus and sorafenib, Andrew X. Zhu led the randomized EVOLVE-1 trial [53] with HCC patients who were not treated with sorafenib. The results, however, fell short of expectations. The everolimus group showed a median PFS of only 7.6 months and a median OS of 3.0 months. Moreover, OS was not improved after treatment with everolimus among patients with liver cancer for whom sorafenib was ineffective or who were intolerant of sorafenib. Everolimus has also been evaluated in a phase III study as a second-line treatment for HCC, though with negative results in an unselected patient population [54]. Thus far, everolimus has not yet been FDA approved for the treatment of HCC.

### C-met inhibitors

C-Met is a proto-oncogene, and the protein becomes phosphorylated upon binding of hepatocyte growth factor. This phosphorylation activates a series of downstream signaling pathways, leading to cell proliferation and survival, cytoskeleton reorganization, cell migration and invasion, and vascular regeneration. This pathway is closely related to the occurrence and development of tumor, and the growth of tumor cells can be inhibited by inhibiting expression of c-Met. Indeed, a previous study demonstrated that overexpression of c-Met is an independent risk factor for poor prognosis in HCC patients [55]. Therefore, c-Met may constitute an alternative molecular target for the development of advanced HCC therapy.

#### Tivantinib (ARQ197)

Tivantinib is an effective small molecule c-Met receptor tyrosine kinase inhibitor. In a phase II trial [56], patients with advanced HCC and Child-Pugh A liver function in the c-Met high-expression group received second-line tivantinib therapy and exhibited a PFS of 2.7 months, which was significantly longer than the 1.4 months observed in the placebo group. Furthermore, Rimassa et al. carried out a phase III study [57] to evaluate the efficacy and safety of tivantinib as a second-line therapy for HCC patients with high Met expression. According to the results, the OS in the tivantinib group was 8.4 months, which was lower than that in the placebo group (9.1 months), and tivantinib-treated patients were more prone to severe adverse events. Overall, tivantinib failed to improve the OS of advanced HCC patients with high Met expression after sorafenib treatment.

## Conclusions and future expectations

**Fig. 2:**
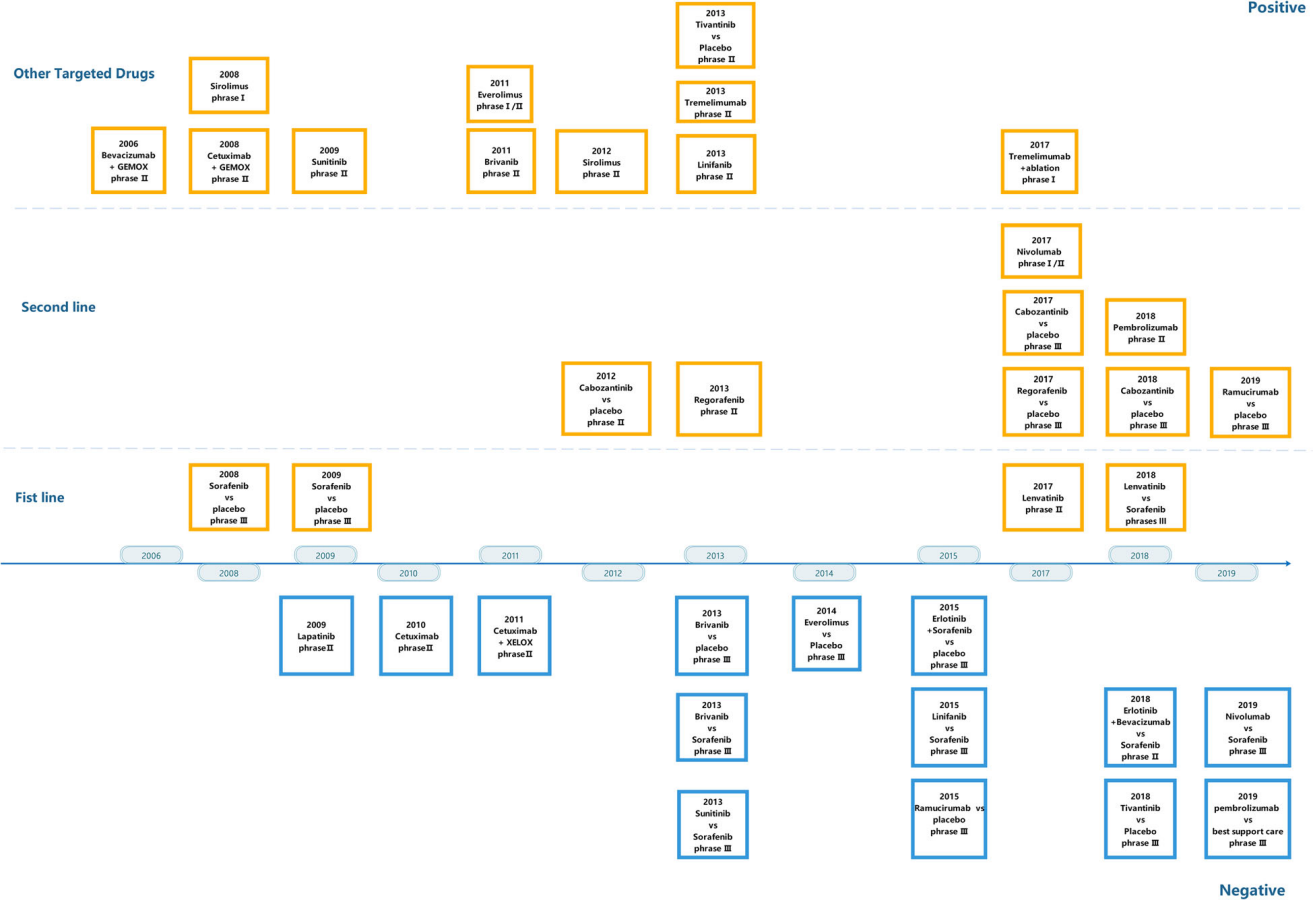
Development and clinical trials of molecular target and immune checkpoint medicines of HCC from 2007 to 2019. Orange: trials with positive results; blue: trials with negative results

Systematic treatment for advanced HCC has changed drastically in the past decade since the introduction of sorafenib as the first small molecule targeting agent in 2007 [58] (Fig. 2). As the pathways and targets closely related to the tumorigenesis and progression of HCC have been revealed, novel molecular targeted therapy agents are constantly being developed and tested, with great expectations of treatment for advanced HCC. However, almost every test of many of these molecular targeted agents during the 10-year period from 2007 to 2016 failed due to a low response rate and high toxicity in phase II or phase III clinical trials. Nonetheless, it is encouraging that in the past 2 years (2017 through 2018), four novel drugs—lenvatinib, regorafenib, cabozantinib, and ramucirumab—have successfully emerged from clinical trials and been recommended for clinical use as alternative or supplements to sorafenib [59]. As recommended by updated BCLC treatment algorithms, lenvatinib is now feasible as an alternative to sorafenib as a first-line treatment for advanced HCC in clinical practice. Regorafenib, cabozantinib, and ramucirumab are appropriate supplements for sorafenib as second-line treatments for patients with advanced HCC who are resistant, have progressed or do not tolerate sorafenib. Recently, with promising outcomes revealed from phase II trials, immune PD-1/PD-L1 checkpoint inhibitors such as nivolumab and pembrolizumab have been applied for HCC treatment [60]. However, in phase III trials, the primary endpoints of OS improvement with nivolumab and pembrolizumab were not statistically significant. Thus, immune PD-1/PD-L1 checkpoint therapy remains to be further investigated.

Finally, the great progress in the number of molecular targeted therapies and immune checkpoint therapy options for advanced HCC will benefit many patients, likely rendering drug selection and sequences challenging. First, combination therapy using targeted treatments with immune checkpoint inhibitors is expected to yield even better effects when these drugs eventually become available. In addition, these new drugs or combination therapies may benefit a wide range of patients in early, intermediate and even advanced stages of HCC as an adjuvant to improve the response rate of chemotherapy, TACE and radiotherapy, to downstage unresectable HCC or to suppress recurrence with high risk. Moreover, biomarkers and alternative predictors, including conventional tumor markers, precise checkpoint targets or pathways, tumor mutational burden (TMB) and circulation tumor cells, remain to be further investigated for precisely identifying patients for appropriate treatment.

## Data Availability

Not applicable.

## Abbreviations

BCLC: Barcelona Clinical Liver cancer
C-MET: Hepatocyte growth factor receptor
CTLA-4: Ytotoxic T lymphocyte associated antigen 4
DCR: Disease control rate
EGFR: Epidermal growth factor receptor
FDA: Food and drug administration
FGFR: Fibroblast growth factor receptor
GEMOX-B: Gemcitabine combined with oxaliplatin and bevacizumab
HCC: Hepatocellular carcinoma
KIT: Stem cell factor receptor
OS: Overall survival
PD-1: Programmed death-1
PDGFR: Platelet-derived growth factor receptor
PD-L1: Programmed death-ligand 1
PFS: Progression-free survival
RET: Glial cell-derived neurotrophic factor receptor
TACE: Transarterial chemoembolization
Tie2: Angiopoietin receptor
TMB: Tumor mutational burden
TTP: Time to progression
VEGFR: Vascular endothelial growth factor receptor
